# Supplementary figures and images for: Phospholipid scramblase 1 (PLSCR1) regulates interferon-lambda receptor 1 (IFN-λR1) and IFN-λ signaling in influenza A virus (IAV) infection
Source: eLife. 2025 Dec 24;14:RP104359. doi: 10.7554/eLife.104359 (PMC12736948; doi:10.7554/eLife.104359)

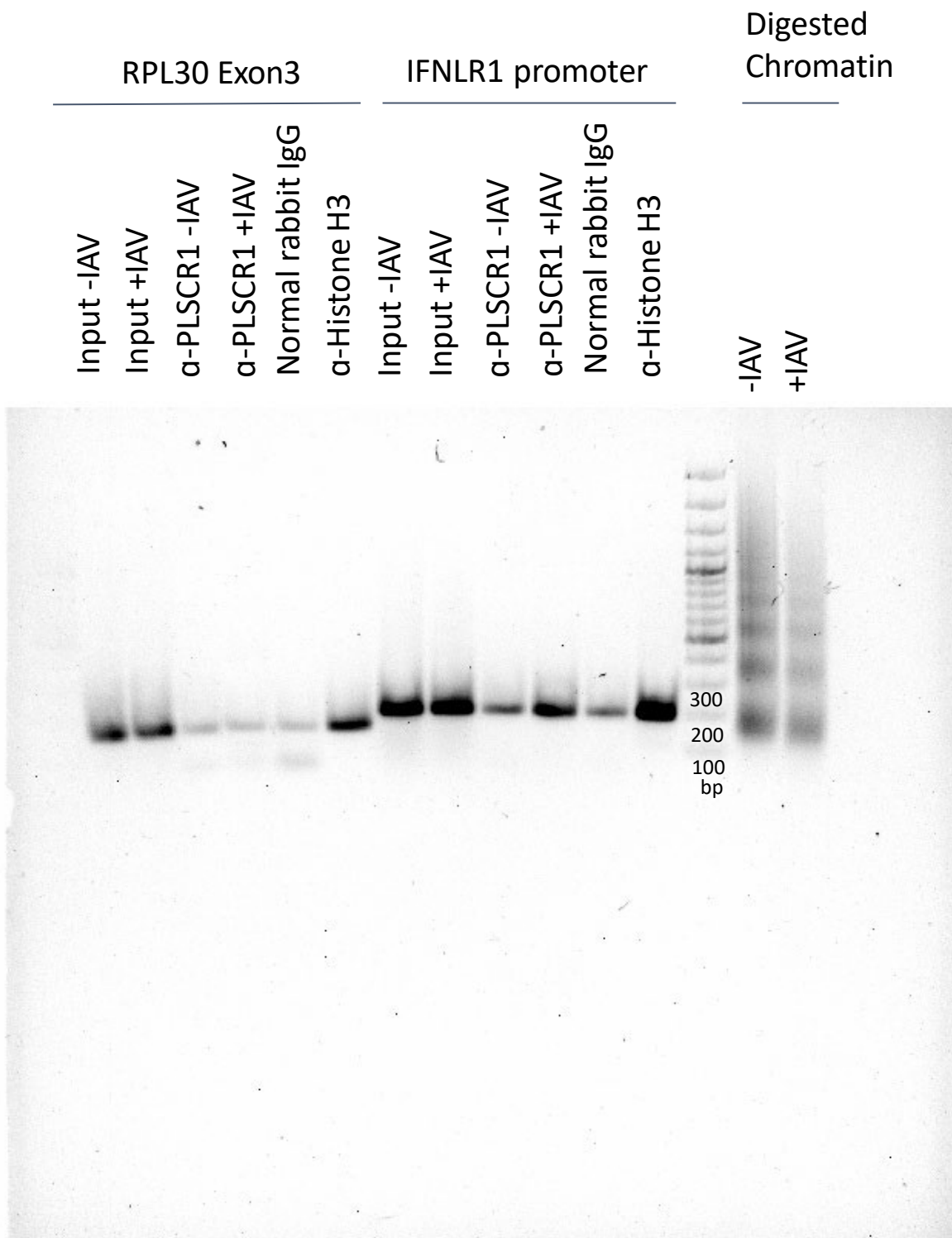

**Figure 3, Source Data 1.** Original gel corresponding to Figure 3, panel H.

Supplement: Figure 3—source data 1. [file elife-104359-fig3-data1.zip › Figure 3, Source Data 1/Figure 3, Source Data 1.pdf]

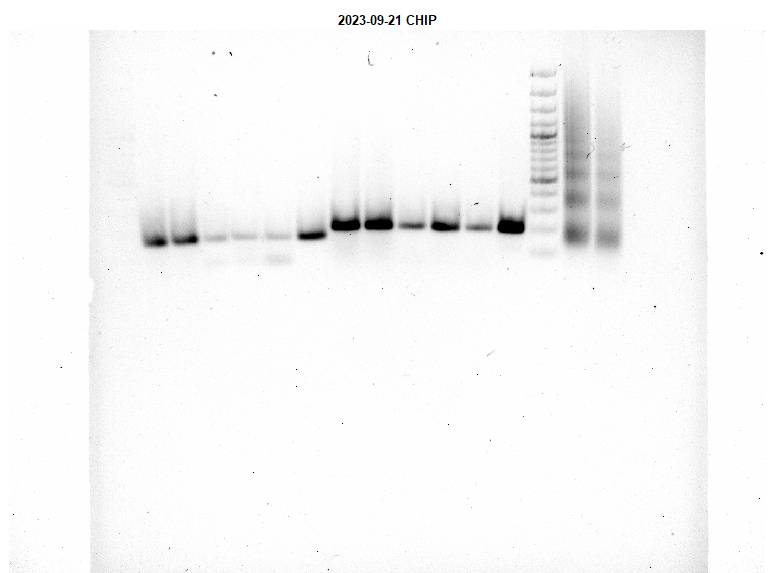

Supplement: Figure 3—source data 2. [file elife-104359-fig3-data2.zip › Figure 3, Source Data 2/source figure 3H.jpg]

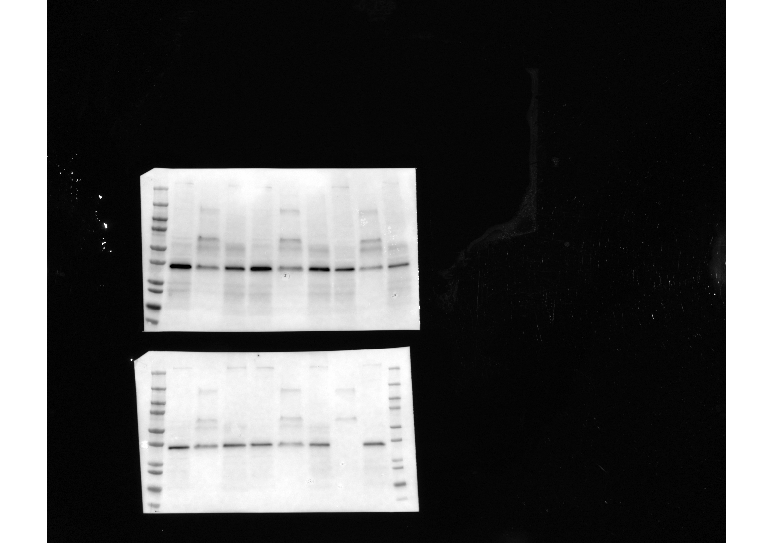

Supplement: Figure 4—source data 2. [file elife-104359-fig4-data2.zip › Figure 4, Source Data 2/source figure 4A-1.jpg]

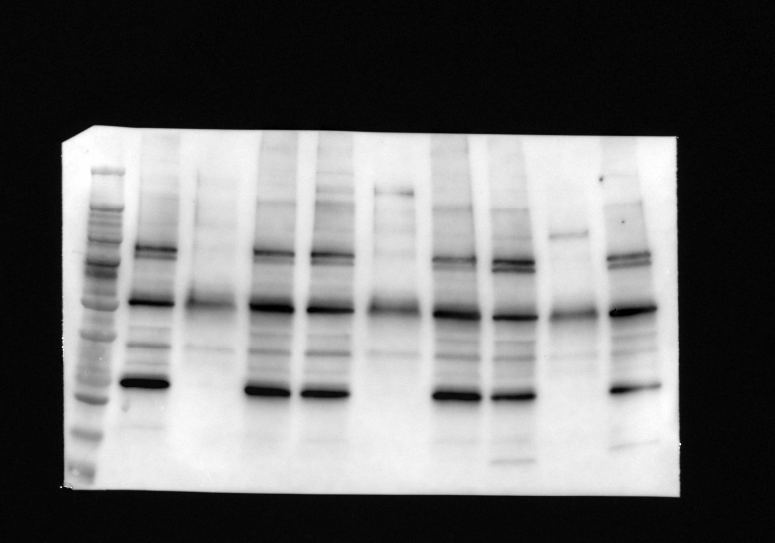

Supplement: Figure 4—source data 2. [file elife-104359-fig4-data2.zip › Figure 4, Source Data 2/source figure 4A-2.tif]

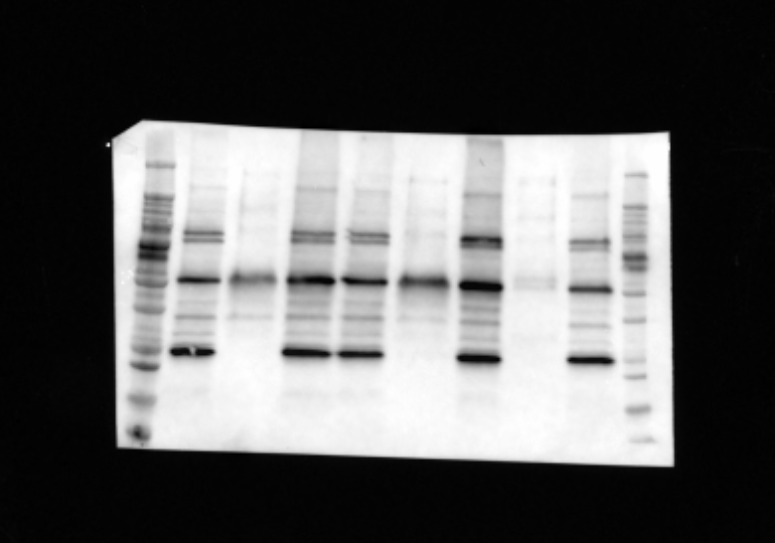

Supplement: Figure 4—source data 2. [file elife-104359-fig4-data2.zip › Figure 4, Source Data 2/source figure 4A-3.tif]

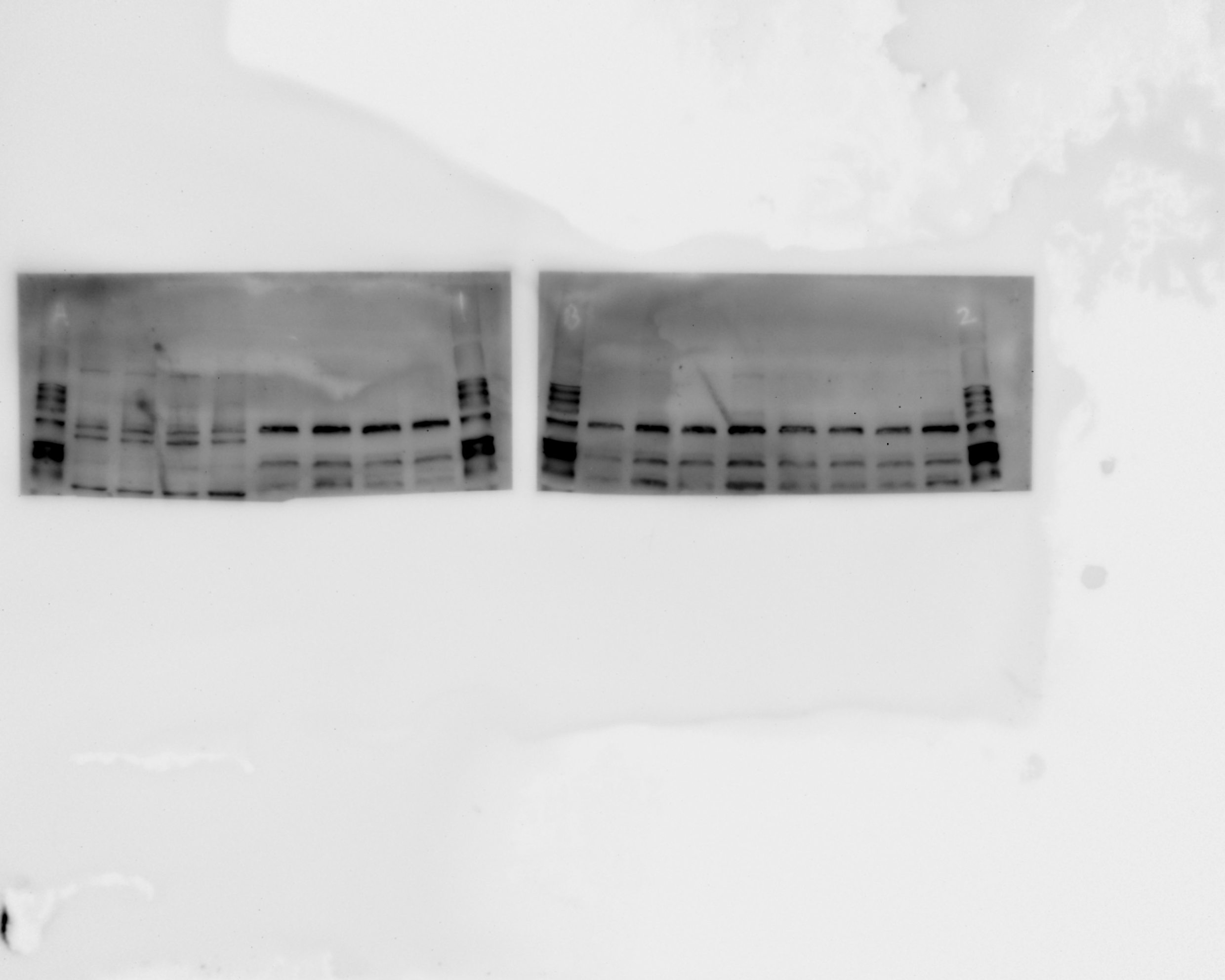

Supplement: Figure 5—source data 2. [file elife-104359-fig5-data2.zip › Figure 5, Source Data 2/source figure 5B-1.tif]

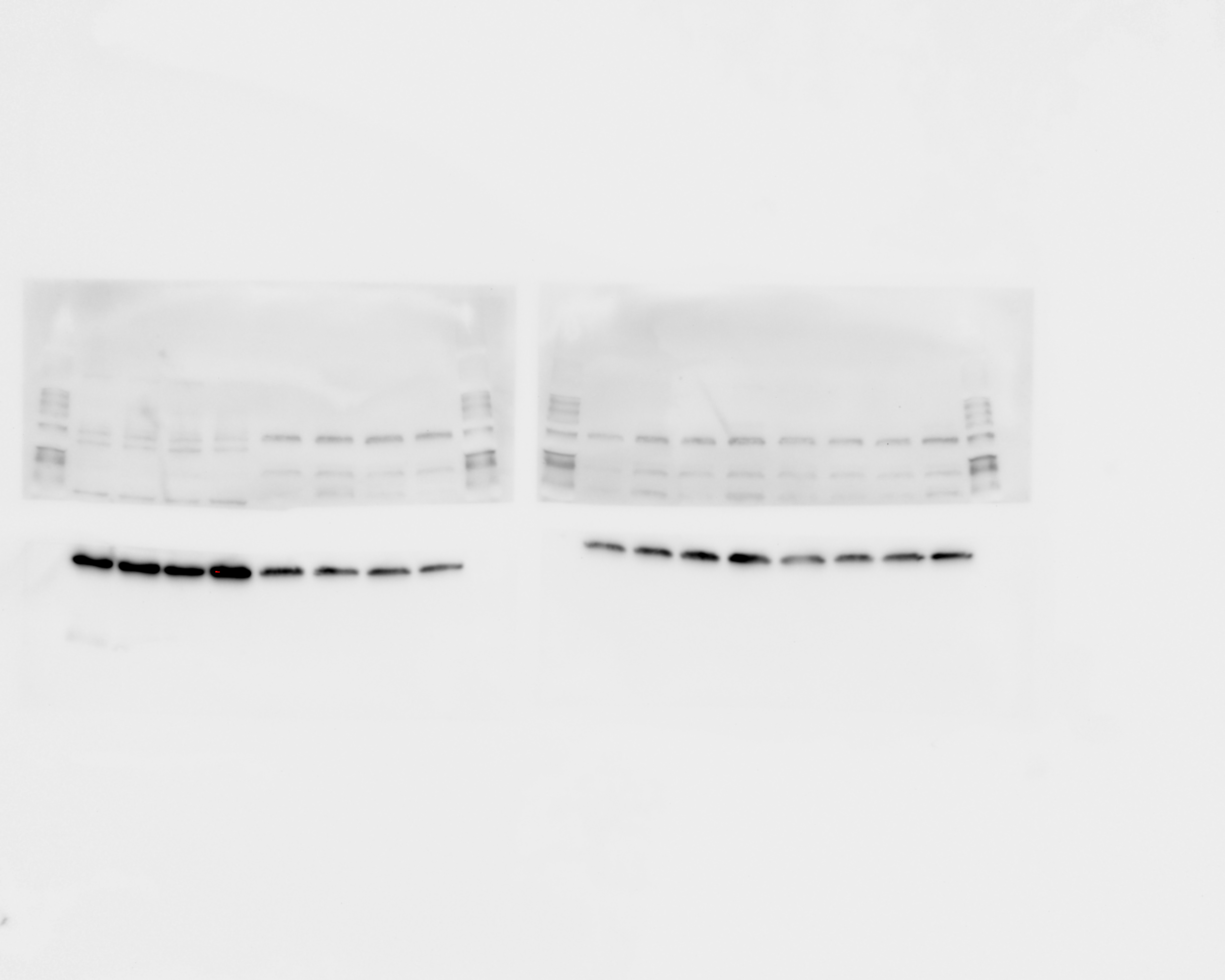

Supplement: Figure 5—source data 2. [file elife-104359-fig5-data2.zip › Figure 5, Source Data 2/source figure 5B-2.tif]

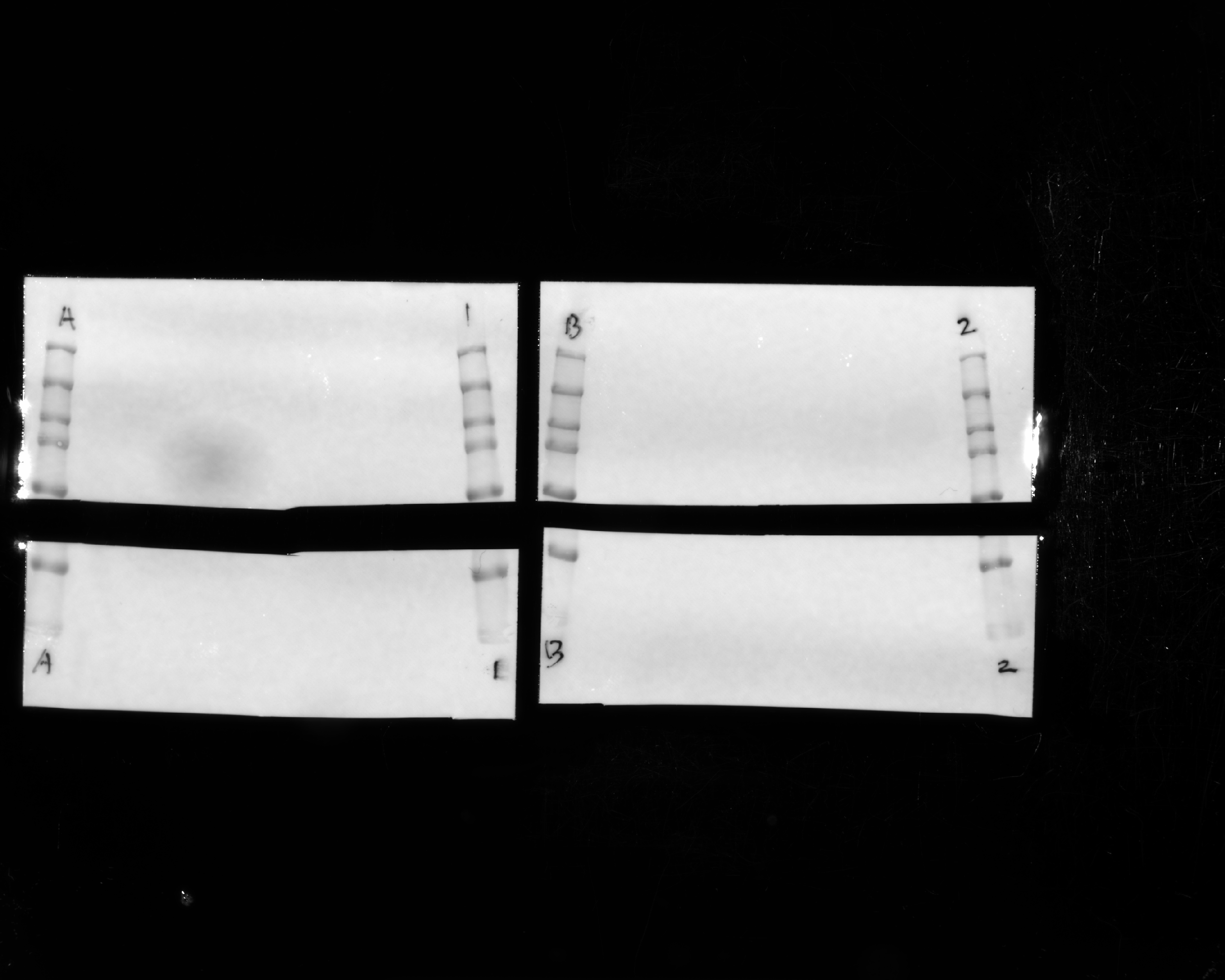

Supplement: Figure 5—source data 2. [file elife-104359-fig5-data2.zip › Figure 5, Source Data 2/source figure 5B-3.tif]

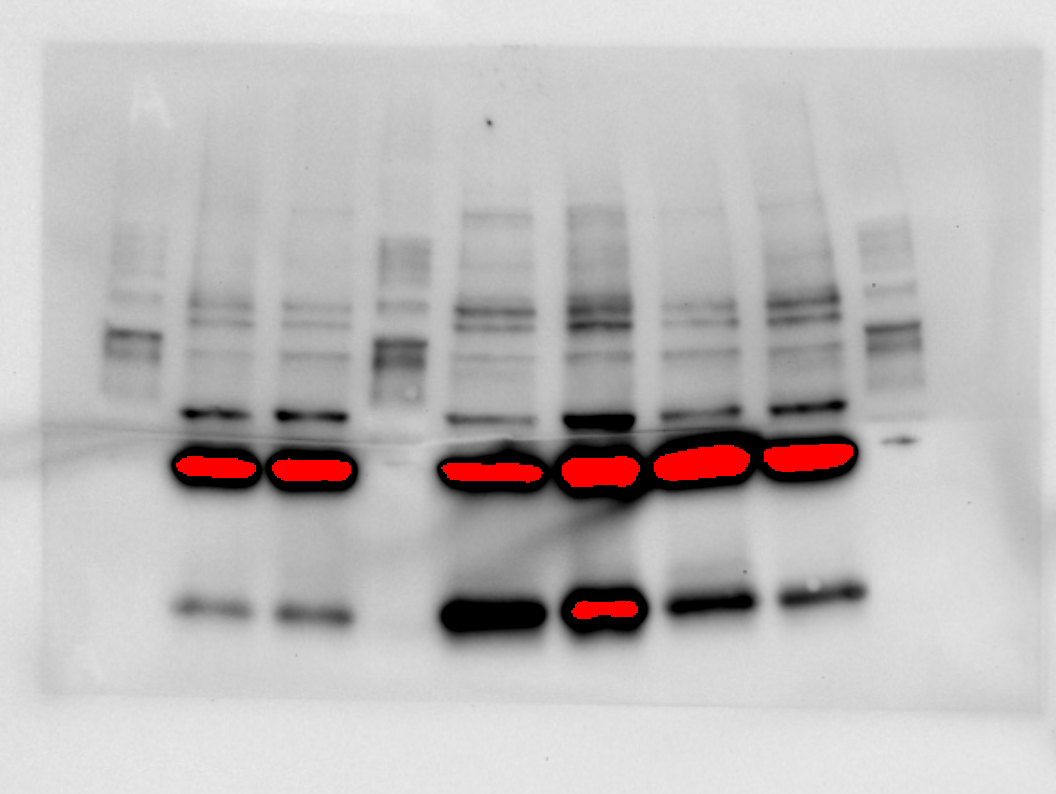

Supplement: Figure 8—source data 2. [file elife-104359-fig8-data2.zip › Figure 8, Source Data 2/source figure 8I-1.tif]

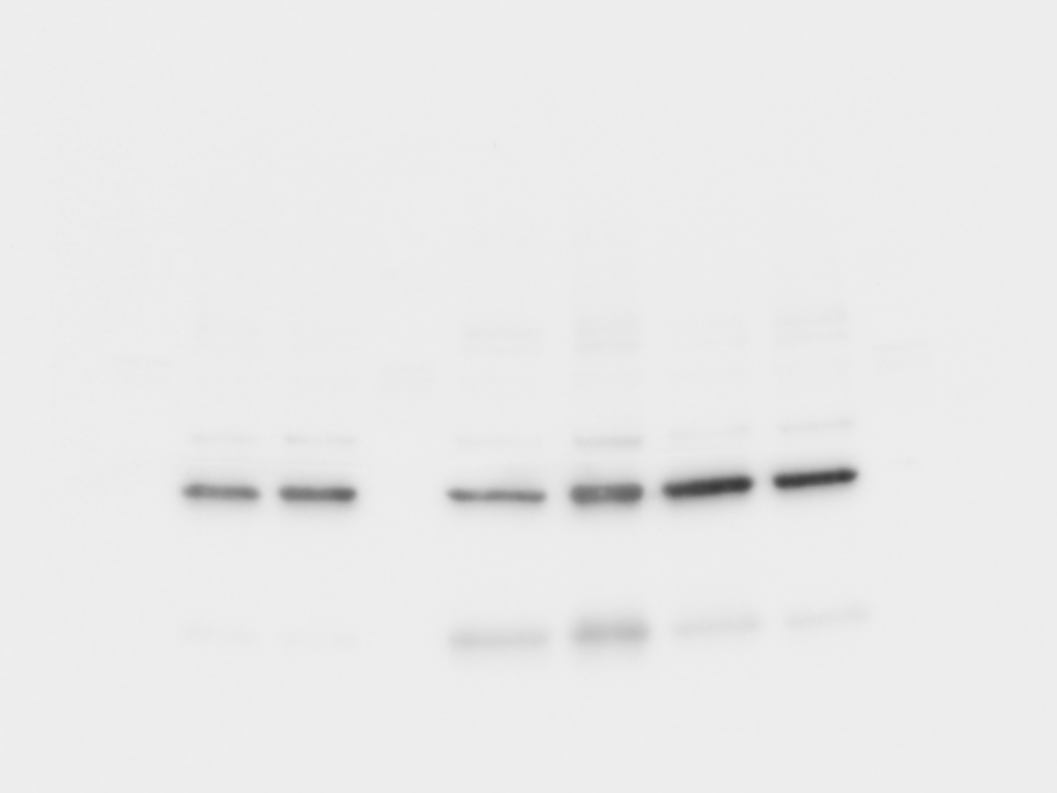

Supplement: Figure 8—source data 2. [file elife-104359-fig8-data2.zip › Figure 8, Source Data 2/source figure 8I-2.tif]

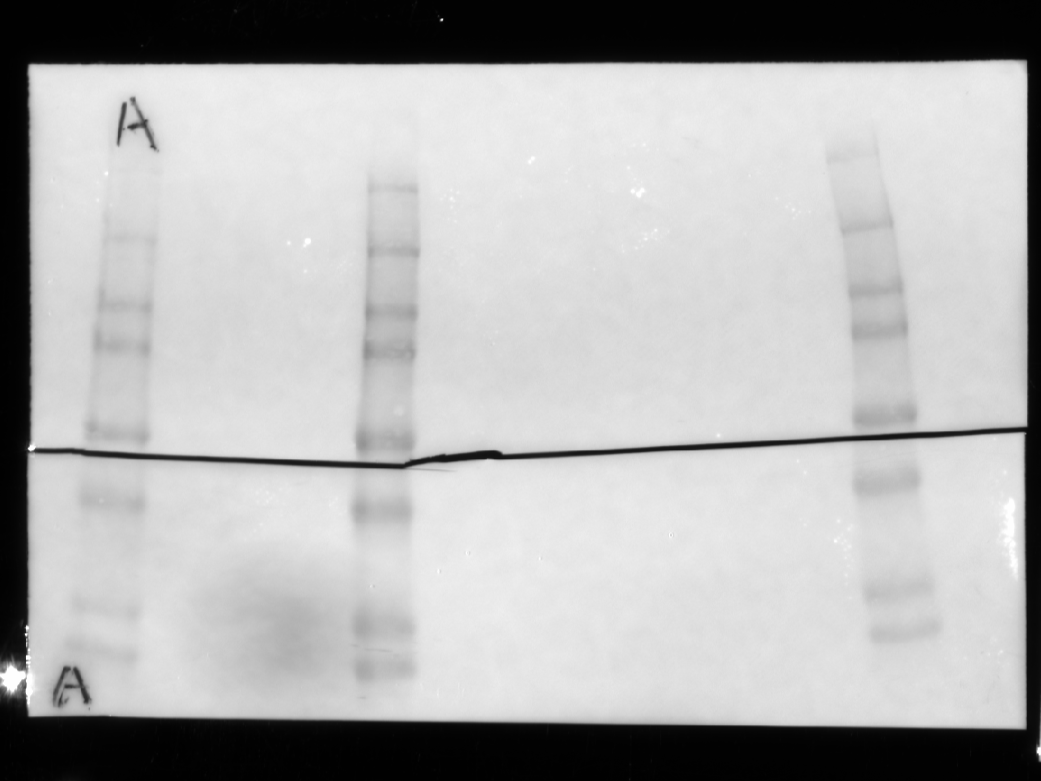

Supplement: Figure 8—source data 2. [file elife-104359-fig8-data2.zip › Figure 8, Source Data 2/source figure 8I-3.tif]
