# Supplementary material for: Phospholipid scramblase 1 (PLSCR1) regulates interferon-lambda receptor 1 (IFN-λR1) and IFN-λ signaling in influenza A virus (IAV) infection
Source: eLife. 2025 Dec 24;14:RP104359. doi: 10.7554/eLife.104359 (PMC12736948; doi:10.7554/eLife.104359)
Supplement: Figure 4—source data 1. — The exposure time was adjusted to visualize Plscr1 (top) or Ifn-λr1 (bottom). [file elife-104359-fig4-data1.zip › Figure 4, Source Data 1/Figure 4, Source Data 1.pdf]

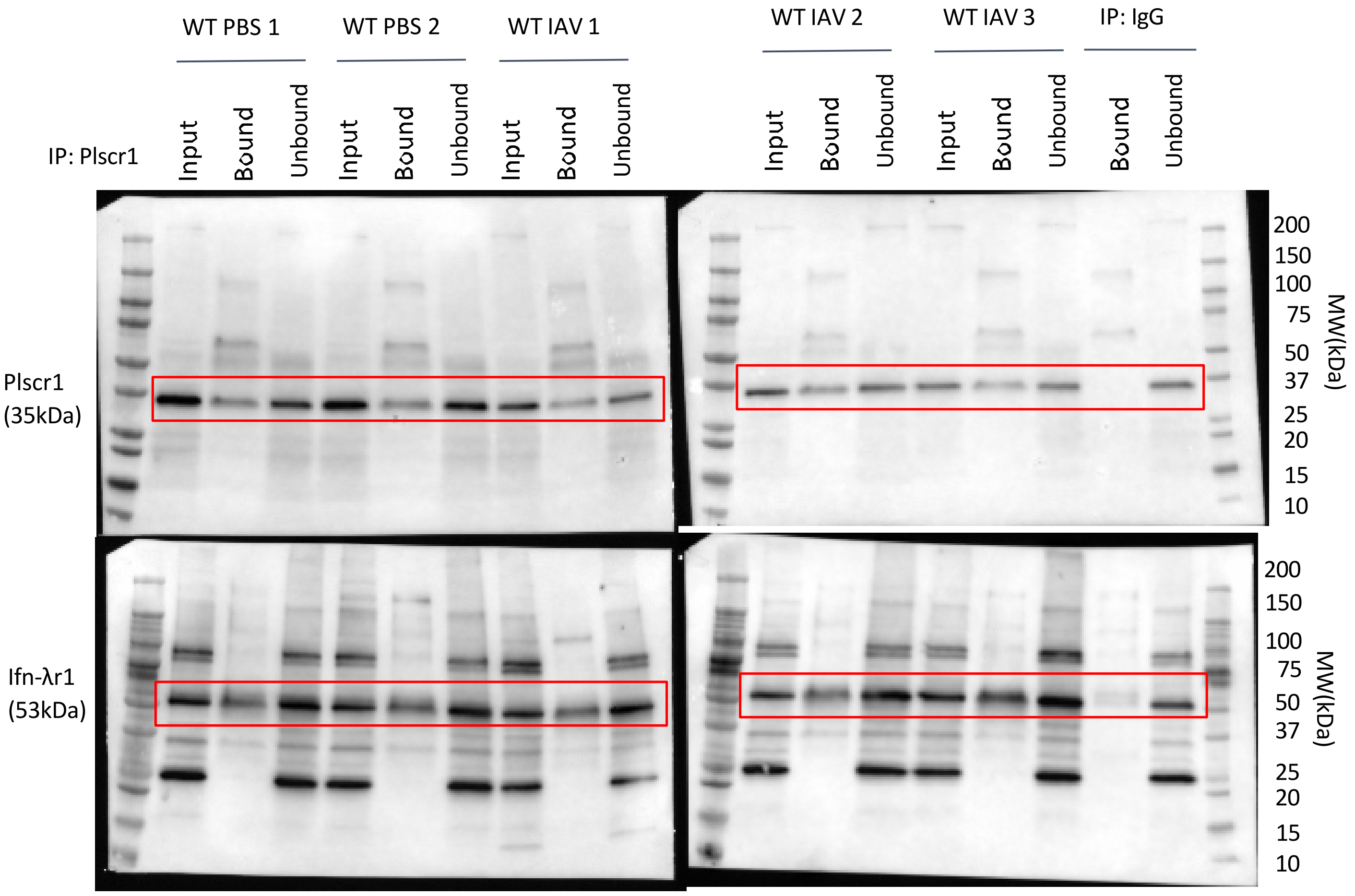

Figure 4, Source Data 1. Original gel corresponding to Figure 4, panel A. The exposure time was adjusted to visualize Plscr1 (top) or Ifn-λr1 (bottom).
