# Supplementary material for: Phospholipid scramblase 1 (PLSCR1) regulates interferon-lambda receptor 1 (IFN-λR1) and IFN-λ signaling in influenza A virus (IAV) infection
Source: eLife. 2025 Dec 24;14:RP104359. doi: 10.7554/eLife.104359 (PMC12736948; doi:10.7554/eLife.104359)
Supplement: Figure 5—source data 1. — The membrane was cut just under 50 kDa marker after transfer. The top part was incubated with α-Ifn-λr1 antibody and the bottom part was incubated with α-β-actin antibody. They were then incubated with corresponding secondary antibodies separately. The exposure time was adjusted to visualize Ifn-λr1 (top) or β-actin (middle). A colorimetric photo was taken to visualize the molecular weight markers (bottom). Lanes 2–5 were from an unrelated experiment. [file elife-104359-fig5-data1.zip › Figure 5, Source Data 1/Figure 5, Source Data 1.pdf]

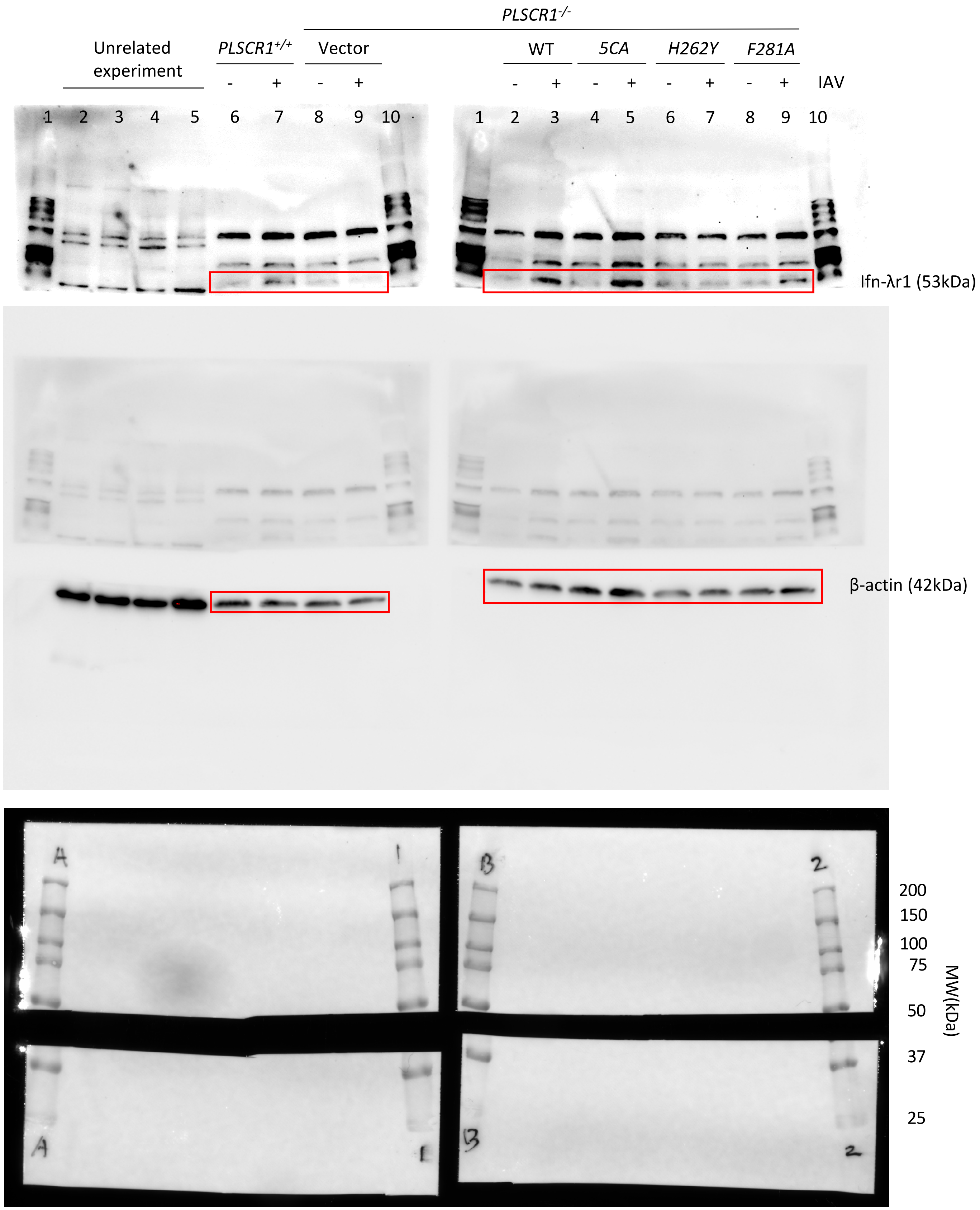

Figure 5, Source Data 1. Original membrane corresponding to Figure 5, panel B. The membrane was cut just under 50 kDa marker after transfer. The top part was incubated with α-Ifn-λr1 antibody and the bottom part was incubated with α-β-actin antibody. They were then incubated with corresponding secondary antibodies separately. The exposure time was adjusted to visualize Ifn-λr1 (top) or β-actin (middle). Colorimetric photo was taken to visualize the molecular weight markers (bottom). Lane 2-5 were from an unrelated experiment.
