# Supplementary material for: Phospholipid scramblase 1 (PLSCR1) regulates interferon-lambda receptor 1 (IFN-λR1) and IFN-λ signaling in influenza A virus (IAV) infection
Source: eLife. 2025 Dec 24;14:RP104359. doi: 10.7554/eLife.104359 (PMC12736948; doi:10.7554/eLife.104359)
Supplement: Supplementary file 1. [file elife-104359-supp1.docx]

| *qRT-PCR primers* | | |
| --- | --- | --- |
| **Gene** | **Primer** | **Sequence (5’-3’)** |
| IAV M gene | Fwd | CATGGAATGGCTAAAGACAAGACC |
|  | Rev | CCATTAAGGGCATTTTGGACA |
| *Plscr1* | Fwd | GCCCAAGTTCACTCTCCAAA |
|  | Rev | GAGCTCAAAGTCAATGTCGG |
| *Ifna* | Fwd | AGTGAGCTGACCCAGCAGAT |
|  | Rev | AGACAGCCTTGCAGGTCATT |
| *Ifnb* | Fwd | CCCTATGGAGATGACGGAGA |
|  | Rev | ACCCAGTGCTGGAGAAATTG |
| *Ifng* | Fwd | ATGGCTATTTCTGGCTGTTACT |
|  | Rev | AATGACGCTTATGTTGTTGCTG |
| *Ifnl2* and *Ifnl3* | Fwd | AGTGGAAGCAAAGGATTG |
|  | Rev | GAGATGAGGTGGGAACTG |
| *Ifnlr1* | Fwd | GACGAGTACAGGCAGCTTCC |
|  | Rev | AGCATTGACCCTTAGGATCTTCTC |
| *Gapdh* | Fwd | AGGTCGGTGTGAACGGATTTG |
|  | Rev | TGTAGACCATGTAGTTGAGGTCA |
| *PLSCR1* | Fwd | CTGACTTCTGAGAAGGTTGC |
|  | Rev | GAATGCTGTCGGTGGATACTG |
| *IFNL1* | Fwd | ACATTGGCAGGTTCAAATCTC |
|  | Rev | TGAGTGACTCTTCCAAGGC |
| *IFNLR1* | Fwd | CAGTGTCCCGAAATACAGCAAG |
|  | Rev | TGTGTCCAGAAAAGTCCAGGGC |
| *IFNLR1* promoter | Fwd | CCGGCCTTGAACTCTCCCT |
|  | Rev | GCGCTCGAAACTCGCCC |
| *GAPDH* | Fwd | TCGTGGAAGGACTCATGACC |
|  | Rev | TCCACCACCCTGTTGCTGTA |

**Supplemental File 1. PCR Primer List.**
