# Supplementary material for: Phospholipid scramblase 1 (PLSCR1) regulates interferon-lambda receptor 1 (IFN-λR1) and IFN-λ signaling in influenza A virus (IAV) infection
Source: eLife. 2025 Dec 24;14:RP104359. doi: 10.7554/eLife.104359 (PMC12736948; doi:10.7554/eLife.104359)
Supplement: Supplementary file 2. [file elife-104359-supp2.docx]

| *scRNA-seq cluster annotations* | | |
| --- | --- | --- |
| **Cluster** | **Cell Type** | **Transcriptional Marker** |
| 0 | AT2 Cells | *Epcam, Sftpc* |
| 1 | Mfap5+ Fibroblasts | *Pdgfra, Mfap5* |
| 2 | Microvascular Endothelial Cells | *Pecam1, Gpihbp1* |
| 3 | Damage-Responsive Fibroblasts | *Pdgfra,* WT*1* |
| 4 | T Cells | *Ptprc, Cd3e* |
| 5 | Activated Microvascular Endothelial Cells | *Pecam1, Gpihbp1, S1pr1* |
| 6 | Airway Smooth Muscle Cells | *Hhip, Acta2* |
| 7 | IAV-Infected Epithelial Cells | *Epcam, Flu* |
| 8 | Microvascular Endothelial Cells | *Pecam1, Gpihbp1* |
| 9 | High Mitochondrial Content Cells |  |
| 10 | Krt8+ Epithelial Cells | *Epcam, Krt8* |
| 11 | AT2 Cells | *Epcam, Sftpc* |
| 12 | Ciliated Epithelial Cells | *Epcam, Foxj* |
| 13 | Club Cells | *Epcam, Scgb3a2* |
| 14 | B Cells | *Ptprc, Cd19* |
| 15 | Myofibroblasts | *Pdgfra, Acta2* |
| 16 | Neutrophils | *Ptprc, Mmp9* |
| 17 | Alveolar Fibroblasts | *Pdgfra, Wnt2* |
| 18 | Epcam+Pecam1+ Cells | *Epcam, Pecam1* |
| 19 | Monocytes | *Ptprc, Ly6c2* |
| 20 | Interferon-Responsive Fibroblasts | *Pdgfra, Bst2* |
| 21 | Mesothelial Cells | *Msln,* WT*1* |
| 22 | Epcam+Col1a2+ EMT Cells | *Epcam, Col1a2* |
| 23 | Interstitial Macrophages | *Ptprc, Cd68, C1qb* |
| 24 | Lymphatic Endothelial Cells | *Pecam1, Prox1* |
| 25 | Aerocytes | *Pecam 1, Car4* |
| 26 | Vascular Smooth Muscle Cells | *Acta2, Pdgfrb, Notch3* |
| 27 | AT1 Cells | *Epcam, Akap5, Ager* |
| 28 | Macrovascular Endothelial Cells | *Pecam1, Vwf* |
| 29 | Ciliated Epithelial Cells | *Epcam, Foxj1* |
| 30 | Alveolar Macrophages | *Ptprc, Cd68, Chil3* |
| 31 | NK Cells | *Ptprc, Klrb1c* |
| 32 | Epcam+Col1a2+ EMT Cells | *Epcam, Col1a2* |
| 33 | Immune Doublets | *Ptprc, Cd3e, Cd19* |
| 34 | Regulatory T cells | *Cd4, ll2ra* |
| 35 | Basophils/Mast Cells | *Ptprc, Fcer1a, Gata2* |
| 36 | Multiplets |  |
| 37 | Platelets |  |

**Supplemental File 2. scRNA-Seq Cluster Annotations.**
